# Bioactive Components in *Moringa Oleifera* Leaves Protect against Chronic Disease

**DOI:** 10.3390/antiox6040091

**Published:** 2017-11-16

**Authors:** Marcela Vergara-Jimenez, Manal Mused Almatrafi, Maria Luz Fernandez

**Affiliations:** 1 Department of Nutrition, Universidad Autonoma de Sinaloa, Culiacan 80019, Mexico; marveji@hotmail.com; 2 Department of Nutritional Sciences, University of Connecticut, Storrs, CT 06269, USA; manal.almatrafi@uconn.edu

**Keywords:** *Moringa Oleifera*, bioactive components, hepatic steatosis, heart disease, diabetes, cancer

## Abstract

*Moringa Oleifera* (*MO*), a plant from the family Moringacea is a major crop in Asia and Africa. *MO* has been studied for its health properties, attributed to the numerous bioactive components, including vitamins, phenolic acids, flavonoids, isothiocyanates, tannins and saponins, which are present in significant amounts in various components of the plant. *Moringa Oleifera* leaves are the most widely studied and they have shown to be beneficial in several chronic conditions, including hypercholesterolemia, high blood pressure, diabetes, insulin resistance, non-alcoholic liver disease, cancer and overall inflammation. In this review, we present information on the beneficial results that have been reported on the prevention and alleviation of these chronic conditions in various animal models and in cell studies. The existing limited information on human studies and *Moringa Oleifera* leaves is also presented. Overall, it has been well documented that *Moringa Oleifera* leaves are a good strategic for various conditions associated with heart disease, diabetes, cancer and fatty liver.

## 1. Introduction

Moringa, a native plant from Africa and Asia, and the most widely cultivated species in Northwestern India, is the sole genus in the family Moringaceae [1]. It comprises 13 species from tropical and subtropical climates, ranging in size from tiny herbs to massive trees. The most widely cultivated species is *Moringa Oleifera (MO)* [1]. *MO* is grown for its nutritious pods, edible leaves and flowers and can be utilized as food, medicine, cosmetic oil or forage for livestock. Its height ranges from 5 to 10 m [1].

Several studies have demonstrated the beneficial effects in humans [2]. *MO* has been recognized as containing a great number of bioactive compounds [3,4] The most used parts of the plant are the leaves, which are rich in vitamins, carotenoids, polyphenols, phenolic acids, flavonoids, alkaloids, glucosinolates, isothiocyanates, tannins and saponins [5]. The high number of bioactive compounds might explain the pharmacological properties of *MO* leaves. Many studies, in vitro and in vivo, have confirmed these pharmacological properties [5].

The leaves of *MO* are mostly used for medicinal purposes as well as for human nutrition, since they are rich in antioxidants and other nutrients, which are commonly deficient in people living in undeveloped countries [6]. *MO* leaves have been used for the treatment of various diseases from malaria and typhoid fever to hypertension and diabetes [7].

The roots, bark, gum, leaf, fruit (pods), flowers, seed, and seed oil of *MO* are reported to have various biological activities, including protection against gastric ulcers [8], antidiabetic [9], hypotensive [10] and anti-inflammatory effects [11]. It has also been shown to improve hepatic and renal functions [12] and the regulation of thyroid hormone status [13]. *MO* leaves also protect against oxidative stress [14], inflammation [15], hepatic fibrosis [16], liver damage [17], hypercholesterolemia [18,19], bacterial activity [20], cancer [14] and liver injury [21].

## 2. Bioactive Components in *Moringa Oleifera*

### 2.1. Vitamins

Fresh leaves from *MO* are a good source of vitamin A [22]. It is well established that vitamin A has important functions in vision, reproduction, embryonic growth and development, immune competence and cell differentiation [23]. *MO* leaves are a good source of carotenoids with pro-vitamin A potential [24].

*MO* leaves also contain 200 mg/100 g of vitamin C, a concentration greater than what is found in oranges [22,25]. *MO* leaves also protect the body from various deleterious effects of free radicals, pollutants and toxins and act as antioxidants [26]. *MO* fresh leaves are a good source of vitamin E, with concentrations similar to those found in nuts [21]. This is important because vitamin E not only acts as an antioxidant, but it has been shown to inhibit cell proliferation [27].

### 2.2. Polyphenols

The dried leaves of *MO* are a great source of polyphenol compounds, such as flavonoids and phenolic acids.

Flavonoids, which are synthesized in the plant as a response to microbial infections, have a benzo-γ-pyrone ring as a common structure [28,29]. Intake of flavonoids has been shown to protect against chronic diseases associated with oxidative stress, including cardiovascular disease and cancer. *MO* leaves are a good source of flavonoids [30].

The main flavonoids found in *MO* leaves are myrecytin, quercetin and kaempferol, in concentrations of 5.8, 0.207 and 7.57 mg/g, respectively [31,32].

Quercetin is found in dried *MO* leaves, at concentrations of 100 mg/100 g, as quercetin-3-*O*-β-d-glucoside (iso-quercetin or isotrifolin) [33,34]. Quercetin is a strong antioxidant, with multiple therapeutic properties [35]. It has hypolipidemic, hypotensive, and anti-diabetic properties in obese Zucker rats with metabolic syndrome [36]. It can reduce hyperlipidemia and atherosclerosis in high cholesterol or high-fat fed rabbits [37,38]. It can protect insulin-producing pancreatic β cells from *Streptozotocin (STZ)* induced oxidative stress and apoptosis in rats [39].

Phenolic acids are a sub-group of phenolic compounds, derived from hydroxybenzoic acid and hydroxycinnamic acid, naturally present in plants, and these compounds have antioxidant, anti-inflammatory, antimutagenic and anticancer properties [40,41]. In dried leaves, Gallic acid is the most abundant, with a concentration of 1.034 mg/g of dry weight. The concentration of chlorogenic and caffeic acids range from 0.018 to 0.489 mg/g of dry weight and 0.409 mg/g of dry weight, respectively [42,43].

Chlorogenic acid (CGA) is an ester of dihydrocinnamic acid and a major phenolic acid in *MO* [44]. CGA has a role in glucose metabolism. It inhibits glucose-6-phosphate translocase in rat liver, reducing hepatic gluconeogenesis and glycogenolysis [45]. CGA has also been found to lower post-prandial blood glucose in obese Zucker rats [46] and to reduce the glycemic response in rodents [47]. CGA has anti-dyslipidemic properties, as it reduces plasma total cholesterol and triglycerides (TG) in obese Zucker rats or mice fed a high fat diet [48] and reverses STZ-induced dyslipidemia in diabetic rats [41].

### 2.3. Alkaloids, Glucosinolates and Isothiocyonates

Alkaloids are a group of chemical compounds, which contain mostly basic nitrogen atoms. Several of these compounds, including *N*,α-l-rhamnopyranosyl vincosamide, phenylacetonitrile pyrrolemarumine,4′-hydroxyphenylethanamide-α-l-rhamnopyranoside and its glucopyranosyl derivative, have been isolated from *Moringa Oleifera* leaves [49,50].

Glucosinolates are a group of secondary metabolites in plants [51]. Both glucosinolates and isothiocyanates have been found to have important health-promoting properties [52].

### 2.4. Tannins

Tannins are water-soluble phenolic compounds that precipitate alkaloids, gelatin and other proteins. Their concentrations in dried leaves range between 13.2 and 20.6 g tannin/kg [53] being a little higher in freeze-dried leaves [54]. Tannins have been reported to have anti-cancer, antiatherosclerotic, anti-inflammatory and anti-hepatoxic properties [55].

### 2.5. Saponins

*MO* leaves are also a good source of saponins, natural compounds made of an isoprenoidal-derived aglycone, covalently linked to one or more sugar moieties [56]. The concentrations of saponins in *MO* freeze-dried leaves range between 64 and 81 g/kg of dry weight [57]. Saponins have anti-cancer properties [58].

## 3. Effects of *Moringa Oleifera* on the Prevention of Chronic Disease

### 3.1. Hypolipidemic Effects

Many bioactive compounds found in *MO* leaves may influence lipid homeostasis. Phenolic compounds, as well as flavonoids, have important roles in lipid regulation [59]. They are involved in the inhibition of pancreatic cholesterol esterase activity, thereby reducing and delaying cholesterol absorption, and binding bile acids, by forming insoluble complexes and increasing their fecal excretion, thereby decreasing plasma cholesterol concentrations [60]. The extracts of *MO* have shown hypolipidemic activity, due to inhibition of both lipase and cholesterol esterase, thus showing its potential for the prevention and treatment of hyperlipidemia [61].

*MO* has a strong effect on lipid profile through cholesterol reducing effects. Cholesterol homeostasis is maintained by two processes: cholesterol biosynthesis, in which 3-hydroxymethyl glutaryl CoA (HMG-Co-A) reductase catalyzes the rate limiting process and cholesterol absorption of both dietary cholesterol and cholesterol cleared from the liver through biliary secretion. The activity of HMG-CoA reductase was depressed by the ethanolic extract of *MO*, further supporting its hypolipidemic action [62]. *Moringa Oleifera (MO)* leaves also contain the bioactive β-sitosterol, with documented cholesterol lowering effects, which might have been responsible for the cholesterol lowering action in plasma of high fat fed rats [18].

Saponins, found in *MO* leaves, prevented the absorption of cholesterol, by binding to this molecule and to bile acids, causing a reduction in the enterohepatic circulation of bile acids and increasing their fecal excretion [9]. The increased bile acid excretion is offset by enhanced bile acid synthesis from cholesterol in the liver, leading to the lowering of plasma cholesterol [9].

### 3.2. Antioxidant Effects

Due to the high concentrations of antioxidants present in *MO* leaves [14,63,64], they can be used in patients with inflammatory conditions, including cancer, hypertension, and cardiovascular diseases [17,65]. The β carotene found in *MO* leaves has been shown to act as an antioxidant. The antioxidants have the maximum effect on the damage caused by free radicals only when they are ingested in combination. A combination of antioxidants found in *MO* leaves was proven to be more effective than a single antioxidant, possibly due to synergistic mechanisms and increased antioxidant cascade mechanisms [22,66,67]. A recent study in children demonstrated that *MO* leaves could be an important source of vitamin A [68].

The extract of *MO* leaves also contains tannins, saponins, flavonoids, terpenoids and glycosides, which have medicinal properties. These compounds have been shown to be effective antioxidants, antimicrobial and anti-carcinogenic agents [69,70]. Phenolic compounds are known to act as primary antioxidants [71], due to their properties for the inactivation of lipid free radicals or prevention of the decomposition of hydroperoxides into free radicals, due to their redox properties. These properties play a key role in neutralizing free radicals, quenching singlet or triplet oxygen, or decomposing peroxides [72,73].

The radical scavenging and antioxidant activities of the aqueous and aqueous ethanol extracts of freeze-dried leaves of *MO*, from different agro-climatic regions, were investigated by Siddhuraju and Becker [74]. They found that different leaf extracts inhibited 89.7–92.0% of peroxidation of linoleic acid and had scavenging activities on superoxide radicals in a dose-dependent manner in the β-carotene-linoleic acid system. Iqbal and Bhanger [75] showed that the environmental temperature and soil properties have significant effects on antioxidant activity of *MO* leaves.

### 3.3. Anti-Inflammatory and Immunomodulatory Effect

The extract of *MO* leaves inhibited human macrophage cytokine production (tumor necrosis factor alpha (TNF-α), interleukin-6 (IL-6) and IL-8), which were induced by cigarette smoke and by lipopolysaccharide (LPS) [76]. Further, Waterman et al. [77] reported that both *MO* concentrate and isothiocyanates decreased the gene expression and production of inflammatory markers in RAW macrophages.

The extracts of *MO* leaves stimulated both cellular and humoral immune responses in cyclophosphamide-induced immunodeficient mice, through increases in white blood cells, percent of neutrophils and serum immunoglobulins [78,79]. In addition, quercetin may have been involved in the reduction of the inflammatory process by inhibiting the action of neutral factor kappa-beta (NF-kβ) and subsequent NF-kB-dependent downstream events and inflammation [80]. Further, fermentation of *MO* appears to enhance the anti-inflammatory properties of *MO* [81]. C57BL/6 mice, fed for 10 weeks with distilled water, fermented and non-fermented *MO* [81]. Investigators reported decreases in the mRNA levels of inflammatory cytokines and reductions in endoplasmic reticulum stress in those animals fed the fermented product.

### 3.4. Hepato-Protective Effects

The methanol extract of *MO* leaves has a hepatoprotective effect, which might be due to the presence of quercetin [14,67]. *MO* leaves had substantial effects on the levels of aspartate amino transferase (AST), alanine amino transferase (ALT) and alkaline phosphatase (ALP), in addition to reductions in lipids and lipid peroxidation levels in the liver of rats [18].

*MO* leaves have been shown to reduce plasma ALT, AST, ALP and creatinine [82,83] and to ameliorate hepatic and kidney damage induced by drugs. In rats, co-treated with *MO* leaves and NiSO4, in order to induce nephrotoxicity, similar findings were observed [84]. Also, Das et al. [75] observed the same reductions in hepatic enzymes in rats fed a high fat diet, in combination with *MO* leaves. Also, the administration of the extract of *MO* leaves in mice was followed by decreases in serum ALT, AST, ALP, and creatinine [85,86]. In guinea pigs, treatment of *MO* leaves prevented non-alcoholic fatty liver disease (NAFLD) in a model of hepatic steatosis, as measured by lower concentrations of hepatic cholesterol and triglycerides in animals treated with *MO* compared to controls [87]. This lowering of hepatic lipids was associated with lower inflammation and expression of genes involved in lipid uptake and inflammation [87]. Further, the *MO* treated guinea pigs had lower concentrations of plasma ASP. In contrast, *MO* leaves did not reduce the inflammation of lipid accumulation in the adipose tissue of guinea pigs [88].

### 3.5. Anti-Hyperglycemic (Antidiabetic) Effect

Many compounds found in *MO* leaves might be involved in glucose homeostasis. For example, isothiocyanates have been reported to reduce insulin resistance as well as hepatic gluconeogenesis [89,90]. Phenolic acids and flavonoids affect glucose homeostasis, influencing β-cell mass and function, and increasing insulin sensitivity in peripheral tissues [91,92]. Phenolic compounds, flavonoids and tannins also inhibit intestinal sucrase and to a certain extent, pancreatic α-amylase activities [56].

The beneficial activities of MO leaves on carbohydrate metabolism have been shown by different mechanisms, including preventing and restoring the integrity and function of β-cells, increasing insulin activity, improving glucose uptake and utilization [57]. Hypoglycemic and antihyperglycemic activity of the leaves of MO might be due to the presence of terpenoids, which are involved in the stimulation of β-cells and the subsequent secretion of insulin. Also, flavonoids have been shown to play an important role in the hypoglycemic action [93]. In another study, where diabetes was induced peritoneally by injection with streptozotocin, rats were fed the equivalent of 250 mg/kg of MO for 6 weeks, using control and diabetic animals [94]. The groups consuming MO extract had significant decreases in malonaldehyde and improvements in the inflammatory cytokines—TNF-α and IL-6—when compared to control animals [94].

### 3.6. Hypotensive Effects

*MO* leaves contain several bioactive compounds, which have been used for stabilizing blood pressure, including nitrile, mustard oil glycosides and thiocarbamate glycosides. The isolated four pure compounds, niazinin A, niazinin B, niazimicin and niazinin A + B—from ethanol extract of *MO* leaves showed a blood pressure lowering effect in rats, mediated possibly through a calcium antagonist effect [14,95]. A recent study reported that *MO* reduced vascular oxidation in spontaneously hypertensive rats [96].

### 3.7. Effects on Ocular Diseases

The major cause of blindness, which ranges from impaired dark adaptation to night blindness, is vitamin A deficiency. *MO* leaves, pods and leaf powder contain high concentrations of vitamin A, which can help to prevent night blindness and eye problems. Also, consumption of leaves with oils improved vitamin A nutrition and delayed the development of cataracts [14].

### 3.8. Anticancer Effects

*MO* has been studied for its chemopreventive properties and has been shown to inhibit the growth of several human cancer cells [97]. The capacity of *MO* leaves to protect organisms and cells from oxidative DNA damage, associated with cancer and degenerative diseases, has been reported in several studies [98]. Khalafalla et al. [99] found that the extract of *MO* leaves inhibited the viability of acute myeloid leukemia, acute lymphoblastic leukemia and hepatocellular carcinoma cells. Several bioactive compounds, including 4-(α-l-rhamnosyloxy) benzyl isothiocyanate, niazimicin and β-sitosterol-3-*O*-β-d-glucopyranoside present in *MO*, may be responsible for its anti-cancer properties [100]. *MO* leaf extract has also been proven to be efficient in pancreatic and breast cancer cells [98,99].

In pancreatic cells, *MO* was shown to contain the growth of pancreatic cancer cells, by inhibiting NF-ĸB signaling as well as increasing the efficacy of chemotherapy, by enhancing the effect of the drug in these cells [101]. In breast cancer cells, the antiproliferative effects of *MO* were also demonstrated [102]. A recent study by Abd-Rabou et al. [103] evaluated the effects of various extracts from *Moringa Oleifera*, including leaves and roots, and preparations of nanocomposites of these compounds against HepG, breast MCF7 and colorectal HCT116/Caco2 cells. All these preparations were effective on their cytotoxic impact, as measured by apoptosis [103]. Several animal studies have also confirmed the efficacy of *Moringa Oleifera* leaves in preventing cancer in rats with hepatic carcinomas induced by diethyl nitrosamine [104] and in suppressing azoxymethane-induced colon carcinogenesis in mice [105]. A list of some bioactive components present in MO leaves, their postulated actions in the animal model used, their protection against a specific disease and the corresponding reference are presented in Table 1.

### 3.9. Protection Against Alzheimer’s Disease (AD) and Parkinson’s Disease (PD)

It is recognized that the monoaminegistic system has a modulatory role in memory processing and that this system is disturbed by AD [106]. Some plants, including *MO*, have been demonstrated to enhance memory by nootropics activity and protect against the oxidative stress present in AD [107] Ganguly et al. [108] have an established model for AD involving the infusion of colchicine into the brain of rats and they demonstrated that *MO* led to the alteration of brain monoamines and electrical patterns. A recent study was conducted to evaluate the effects of an isothiocynate isolated from *MO*, both in a mouse model of PD and in RAW 264.7 macrophages stimulated with LPS [109]. Results demonstrated great efficacy of a bioactive compound in *MO*, which results from myrosinase hydrolysis in favorably modulating the inflammatory and apoptotic pathways as well as oxidative stress [109].

## 4. Conclusions

**Figure 1:**
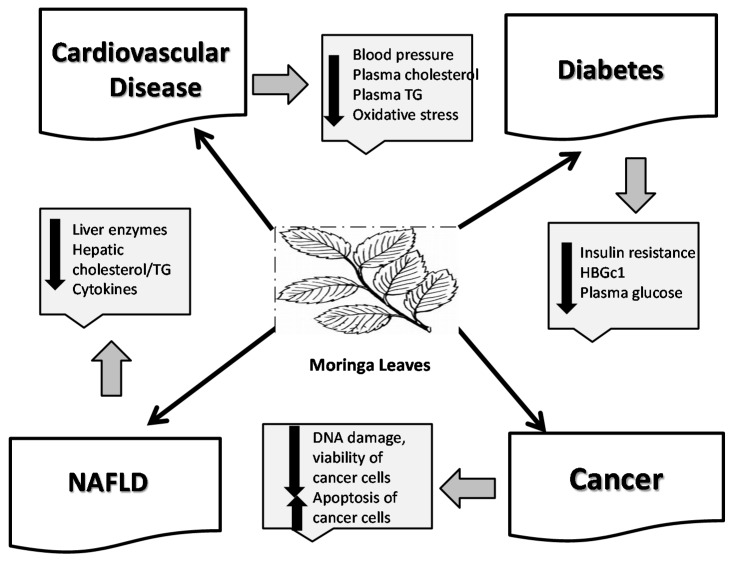
Protective effects of *MO* leaves against chronic diseases: **cardiovascular disease**, by lowering plasma lipids including triglycerides (TG) [45,60] decreasing blood pressure [92] and reducing oxidative stress [73]; **diabetes**, by lowering plasma glucose [61], reducing insulin resistance [89] and increasing β cell function [90]; **NAFLD**, by reducing hepatic lipids [82,87], reducing liver enzymes [82,83,88] and decreasing hepatic inflammation [88] and **cancer**, by reducing DNA damage [97], viability of cancer cells [99,100] and increasing apoptosis [104,105].

In summary, there are a number of animal studies documenting the effects of *MO* leaves in protecting against cardiovascular disease, diabetes, NAFLD, Alzheimer’s, hypertension and others, due the actions of the bioactive components in preventing lipid accumulation, reducing insulin resistance and inflammation. Additional studies in humans, including clinical trials are needed to confirm these effects of *MO* on chronic diseases. In addition, some studies have found that the compounds in *MO* may also protect against Alzheimer’s disease and Parkinson’s disease. A summary of the effects of the bioactive component of *MO* leaves in protecting against these conditions is shown in Figure 1.

## Figures and Tables

**Table 1:** Bioactive Components in *Moringa Oleifera* and their Positive Effects on Chronic Disease.

Compounds	Postulated Function	Model Used	Disease Protection	References
Flavonoids: Quercitin	Hypolipidemic and anti-diabetic properties	Zucker rat	Diabetes	[36]
	Lower hyperlipidemia	Rabbits	Atherosclerosis	[37,38]
	Decrease expression of DGAT	Guinea Pigs	NAFLD	[80]
	Inhibition of cholesterol esterase and α-glucosidase	In vitro study	Cardiovascular disease and Diabetes	[60]
	Inhibits activation of NF-kB	High fat fed Mice	Cardiovascular disease	[74]
Chlorogenic Acid	Glucose lowering effect	Diabetic rats	Diabetes	[45]
	Cholesterol lowering in plasma and liver	Zucker rat	Cardiovascular disease	[46]
	Decrease expression of CD68, SERBP1c	Guinea pigs	NAFLD	[87]
	Anti-obesity properties	High-fat induced obesity rats	Obesity	[49]
	Inhibit enzymes linked to T2D		Diabetes	[90]
Alkaloids	Cardioprotection	Cardiotoxic-induced rats	Cardiovascular disease	[49]
Tannins	Anti-inflammatory	Rats	Cardiovascular/Cancer	[54]
Isothiocyanates	Decreased expression of inflammatory markers	RAW Macrophages	Cardiovascular disease	[76]
	Reduction in insulin resistance	Mice	Diabetes	[88]
	Inhibition of NF-kB signaling	Cancer breast cells	Cancer	[99]
Β-Sitosterol	Decrease cholesterol absorption	High-fat fed rats	Cardiovascular disease	[18]

Abbreviations used: CD68: cluster of differentiation 68; DGAT: diacyl glycerol transferase; NF-kB: nuclear factor-kB; SRBP1c: sterol regulatory binding protein 1c; T2D: type 2 diabetes; NAFLD: non-alcoholic fatty liver disease.
